# Outputs, cost and efficiency of public sector centres for prevention of mother to child transmission of HIV in Andhra Pradesh, India

**DOI:** 10.1186/1472-6963-8-26

**Published:** 2008-01-31

**Authors:** Lalit Dandona, SG Prem Kumar, YK Ramesh, M Chalapathi Rao, Elliot Marseille, James G Kahn, Rakhi Dandona

**Affiliations:** 1 George Institute for International Health – India, Hyderabad, India; 2 Health Studies Area, Centre for Human Development, Administrative Staff College of India, Hyderabad, India; 3 George Institute for International Health, University of Sydney, Sydney, Australia; 4 School of Public Health, University of Sydney, Sydney, Australia; 5 Faculty of Medicine, University of Sydney, Sydney, Australia; 6 Institute for Health Policy Studies and AIDS Research Institute, University of California, San Francisco, USA

## Abstract

**Background:**

Prevention of mother to child transmission (PMTCT) is an important part of the effort to control HIV. PMTCT services are mostly provided at public sector government hospitals in India. Systematic data on the cost and efficiency of providing PMTCT services in India are not available readily for further planning.

**Methods:**

Cost and output data were collected at 16 sampled PMTCT centres in the south Indian state of Andhra Pradesh using standardized methods. The services provided were analysed, and the relation of unit cost of services with scale was assessed.

**Results:**

In the 2005–2006 fiscal year, 125,073 pregnant women received PMTCT services at the 16 centres (range 2,939 to 20,896, median 5,679). The overall HIV positive rate among those tested was 1.67%. Of the total economic cost, the major components were personnel (47.3%) and recurrent goods (31.7%). For the 16 PMTCT centres, the average economic cost per post-HIV-test counselled pregnant woman was Indian Rupees (INR) 98.9 (US$ 2.23), ranging 2.7-fold from INR 71.4 (US$ 1.61) to INR 189.9 (US$ 4.29). The economic cost per mother-neonate pair who received nevirapine had a higher variation, ranging 41-fold for the 16 centres from INR 4,354 (US$ 98) to INR 179,175 (US$ 4,047), average INR 10,210 (US$ 231), with very high unit cost at some centres where HIV prevalence among pregnant women and the total volume of services were both low. Scale had a significant inverse relation with both of the unit costs, per post-HIV-test counselled pregnant woman and per mother-neonate pair who received nevirapine. In addition, HIV prevalence among pregnant women had a significant inverse relation with unit cost per mother-neonate pair who received nevirapine.

**Conclusion:**

Although the variation between PMTCT centres for unit cost per post-HIV-test counselled pregnant woman was modest that per mother-neonate pair receiving nevirapine was over 40-fold. The extremely high unit cost for each mother-neonate pair receiving nevirapine at some centres suggests that the new approach of combining PMTCT services with voluntary counselling and testing services that has recently been started in India could potentially offer better efficiency.

## Background

Although the estimate of the number of people living with HIV in India has been reduced to less than half recently based on new population-based data, this is still one of the highest numbers for a country in the world [1-4]. The south Indian state of Andhra Pradesh is estimated to have the highest number of HIV infected persons in India, and had the highest HIV positive rate (1.26%) among pregnant women tested at public sector sentinel centres in the sentinel surveillance of 2006 [4]. Prevention of Mother to Child Transmission (PMTCT) of HIV is an important component of the HIV prevention services in this state.

The Reproductive and Child Health survey of 2002–2004 estimated that in Andhra Pradesh 60.9% of the deliveries were institutional, with 22.1% in public sector health facilities and 38.8% in the private sector [5]. PMTCT services are available only at some public sector facilities and therefore the majority of pregnant women are not receiving these services. The PMTCT services were started in Andhra Pradesh at one centre situated in a tertiary hospital attached to a medical college in the year 2000. Subsequently 13 PMTCT centres were started in hospitals attached to a medical college in the year 2002, and 23 PMTCT centres were started in hospitals not attached to a medical college in the year 2003. During the 2005–2006 fiscal year 64 more PMTCT centres were started, mostly at smaller area hospitals and community health centres which are not attached to a medical college. We assessed the cost and efficiency of the PMTCT centres in Andhra Pradesh to inform further planning of HIV prevention services.

## Methods

The methods used in this study were adapted from the multi-country Prevent AIDS Network for Cost Effectiveness Analysis (PANCEA) study [6-9]. Description of the methods relevant for this paper follows.

### Selection of PMTCT centres

At the time of data collection in mid-2006, there were 37 public sector PMTCT centres that had functioned throughout the 2005–2006 fiscal year in Andhra Pradesh. Another 64 PMTCT centres were started towards the end of 2005 but because they had not functioned for the full 2005–2006 fiscal year they were not considered for our sample. Andhra Pradesh has three geographic regions: the northern Telangana region has the state capital and nine other districts, the eastern Coastal region has nine districts, and the southern Rayalseema region has four districts with a population nearly half that in either of the other two regions. We used the three geographic regions of Andhra Pradesh as the strata for sampling. Within each geographic region we selected about 50% of the centres situated in hospitals attached to a medical college and about 50% situated in hospitals not attached to a medical college, so that we could assess the efficiency of different sizes of hospitals – the former are much larger than the latter. Within these two strata, 16 PMTCT centres were randomly sampled to obtain six centres each in the Telangana and Coastal regions and four centres in the Rayalseema region.

### Data collection procedures

Data were collected for the April 2005 – March 2006 fiscal year at the 16 sampled PMTCT centres during June – July 2006. Data collection included a history of the PMTCT centre and detailed cost and output data by month. Formal consent to collect data was obtained from the Andhra Pradesh State AIDS Control Society (APSACS) and the superintendents of the hospitals where the PMTCT centres were located. Data collection at each centre was started with an interview with the PMTCT medical officer/s (a microbiologist and/or a gynaecologist and/or a paediatrician), counsellor(s), and laboratory technician. The interview contained structured open-ended questions regarding the history of the PMTCT centre and operational and community factors that affect the demand and supply of services. After this, cost and output data was collected. Data collection at a PMTCT centre was done by two investigators and lasted for one week. Data were recorded in the field on laptop computers in MS Excel and MS Word files.

### Cost data

Data on both financial and economic cost were collected, the latter being the true resource cost incurred. The cost data were collected under five heads: rentals, personnel, capital goods, recurrent goods and recurrent services. Similar costing methods were used for all the PMTCT centres. To convert Indian Rupees (INR) into United States Dollars (US$) the average exchange rate of INR 44.27 = US$1 was used for the April 2005 – March 2006 fiscal year [10].

All PMTCT centres were located in a parent public sector hospital, and as such no rent was paid. In order to calculate economic cost of the rental equivalent, the floor area occupied by each PMTCT centre was determined, rent rates obtained from three sources in that area for health facilities for the 2005–2006 fiscal year, and the average of these rates applied to the PMTCT area.

Salary cost was computed for all personnel contributing to the work of the PMTCT centre, which included the medical officers, counsellor(s) and laboratory technician. For personnel contributing part time to the PMTCT work, only that proportion of salary was included in the PMTCT salary cost. For example, if the medical officers were contributing only one-third of their time for PMTCT and the rest of the time to teaching and/or providing other clinical services, then only one-third of their salary was included in the personnel cost. Information about the salaries of the personnel was collected from the official records of the accounts section of the hospital of which the PMTCT centre was a part. In addition to the regular salary, if some personnel were paid fringe benefits, these were included in the cost.

Capital goods included furniture, electrical fixtures, air conditioner, air cooler, refrigerator, centrifuge, computer, needle and syringe destroyer, hot air oven, micropipette, weighing machine, infantometer and public address system. If information about the cost of the capital goods was not available from the PMTCT centre or its parent public sector hospital, the market price was determined from retail sellers of these goods. The life of capital goods was assumed to be five years, and therefore, one-fifth of the cost was allocated to the 2005–2006 fiscal year if the good was used for the full year. If a capital good was purchased in the middle of this fiscal year and used only for half the year, the cost allocated for this item was half of the yearly cost. If a capital good, for example refrigerator or centrifuge was also being used for work other than that of the PMTCT centre, a determination was made from the PMTCT centre staff about the proportion of use for PMTCT centre work and that proportional cost was allocated to the PMTCT centre.

Recurrent goods utilised at the PMTCT centres included HIV test kits, nevirapine tablets and syrup, disposable surgical kits, disposable delivery kits, needles and syringes, gloves, sterillium, spirit, sodium hypochlorite solution, distilled water, test tubes, serum containers, droppers, micro-tips, cotton, filter paper, soap, stationery and some miscellaneous items. Several recurrent goods were not directly purchased by the PMTCT centres, for example, the HIV test kits, nevirapine tablets and nevirapine syrup were supplied to the PMTCT centres by APSACS. These goods are purchased in bulk from commercial manufacturers. Information was obtained from the manufacturers about the prices for such bulk purchases during the 2005–2006 fiscal year and these were used as the cost. Attempts were made to get the cost of the other goods from the records of the PMTCT centres, which if available were used for analysis. If not available, three quotations for these goods were obtained from the market for the 2005–2006 fiscal year and the average of these taken as the cost.

Recurrent services included training of staff, cleaning and building maintenance, electricity, water, telephone, waste disposal, photocopying, postage and courier, and some miscellaneous items. The cost for staff training was calculated by including travel fare, per diem, trainer fees, training materials, and training facility cost, information about which was obtained not only from the PMTCT centres, which incurred some of these costs, but also from the APSACS, which incurred many of these costs. The cost of building maintenance was calculated based on the space occupied by the PMTCT centre. Electricity and water costs were based on applying the market rates to the estimated usage. Telephone and other recurrent services costs were calculated based on actual usage.

For financial cost calculations the rentals were excluded because the infrastructure was already in place when the government decided to provide PMTCT services. The salary of the medical officer was excluded because the medical officer is not paid anything extra for providing the PMTCT services. The annualised cost of capital goods provided by the hospital was also excluded because these capital goods were already in place before the PMTCT centres started. However the cost of the recurrent goods and services provided by the hospital were included because that is an additional cost for providing PMTCT services.

### Output data

Detailed data were obtained from the written records of the PMTCT centres regarding the services provided by month for the 2005–2006 fiscal year. These included the number, format and duration of pre- and post-HIV-test counselling sessions; number of HIV tests conducted and results; number of mother-neonate pairs who received nevirapine; number of medical terminations of pregnancy in HIV positive women; number of deliveries conducted in HIV negative and positive women; and number of spouse/partners tested and post-HIV-test counselled. Characteristics of programme operation were ascertained, including client characteristics and types of HIV tests administered.

### Quality control

Quality control measures included comprehensive training of the data collection team in standardised methods including their understanding of all data issues, full back-up and justification for any data recorded, supervision of data collection at each PMTCT centre, thorough review by the study team of the data obtained at each PMTCT centre, and contacting the PMTCT centres again to obtain information about data issues that needed clarification after the review.

### Data analysis

After review of data and clarifications, analysis of the outputs and cost data was done using SPSS and Microsoft Excel softwares. Efficiency of PMTCT centres was assessed for two outputs, economic cost per post-HIV-test counselled pregnant women (which includes information that they and their neonates could get anti-retroviral treatment at the time of delivery if they were HIV positive) and economic cost per mother-neonate pair receiving nevirapine if the mother were HIV positive. The relation between scale and unit costs was analysed using regression fits, separately for the larger PMTCT centres situated in tertiary hospitals attached to a medical college and those situated in hospitals not attached to a medical college, and also combined for these two types of PMTCT centres. The relation between HIV prevalence among pregnant women at the PMTCT centres and the unit cost per mother-neonate pair receiving nevirapine was also assessed using regression analysis. Bivariate regression types available in Microsoft Excel (exponential, linear, logarithmic, polynomial and power) were used, and the relations with the best fit are presented.

## Results

A total of 125,073 pregnant women received post-HIV-test counselling at the 16 PMTCT centres in the fiscal year 2005–2006, ranging from 2,939 to 20,896 in a centre, with a median of 5,679 and a mean of 7,817 (Table 1). The average number of post-HIV-test counselled at the 8 PMTCT centres situated in hospitals attached to a medical college was 11,368 versus 4,266 at the 8 PMTCT centres situated in hospitals not attached to a medical college.

**Table 1:** HIV testing at PMTCT centres in the 2005–2006 fiscal year.

**PMTCT centre serial number**	**Number of HIV tests done in pregnant women**	**Number (%) HIV positive**	**Number of post-HIV-test counselled pregnant women**	**Number (%) tested but did not collect results**	**Number of HIV positive post-test counselled**	**Number (%) of HIV positive who did not collect test results**
**At hospitals attached to medical college**						

2	10605	81 (0.76)	10331	274 (2.58)	77	4 (4.94)
5	11989	372 (3.10)	10039	1950 (16.26)	321	51 (13.71)
7	5615	118 (2.10)	4659	956 (17.03)	100	18 (15.25)
8	9185	125 (1.36)	8726	459 (5.00)	124	1 (0.80)
9	11895	364 (3.06)	11496	399 (3.35)	341	23 (6.32)
12	12842	147 (1.14)	12263	579 (4.51)	136	11 (7.48)
13	21363	253 (1.18)	20896	467 (2.19)	253	0 (0.00)
15	13556	117 (0.86)	12537	1019 (7.52)	108	9 (7.69)

**Sub-total**	**97050**	**1577 (1.62)**	**90947**	**6103 (6.29)**	**1460**	**117 (7.42)**

**At hospitals not attached to medical college**						

1	5078	80 (1.58)	5078	0 (0.00)	80	0 (0.00)
3	3019	17 (0.56)	2963	56 (1.85)	17	0 (0.00)
4	5057	138 (2.73)	4831	226 (4.47)	136	2 (1.45)
6	3011	79 (2.62)	2939	72 (2.39)	77	2 (2.53)
10	3688	36 (0.98)	3631	57 (1.55)	36	0 (0.00)
11	5989	130 (2.17)	5777	212 (3.54)	116	14 (10.77)
14	5843	112 (1.92)	5580	263 (4.50)	109	3 (2.68)
16	3372	31 (0.92)	3327	45 (1.33)	29	2 (6.45)

**Sub-total**	**35057**	**623 (1.78)**	**34126**	**931 (2.66)**	**600**	**23 (3.69)**

**Total**	**132107**	**2200 (1.67)**	**125073**	**7034 (5.32)**	**2060**	**140 (6.36)**

Of all pregnant women tested for HIV, 1.67% were found positive. The HIV positive rate was highest in the coastal Andhra region at 2.65% versus 1.17% in the other two regions of the state. The overall drop-out rate between tests done and post-HIV-test counselled was 5.3%, and that for HIV positive women was somewhat higher at 6.4% (Table 1). The latter was highest in Coastal Andhra (9.4%). The HIV positive rate was similar in PMTCT centres situated in hospitals attached to a medical college and those not attached to a medical college, but the drop-out rates were lower in the latter (Table 1).

The total number of deliveries at the 16 PMTCT centres in the 2005–2006 fiscal year was 91,312, of which 1,322 (1.45%) were in HIV positive women (Table 2). The PMTCT centres provide nevirapine to the HIV positive mothers and neonates to prevent vertical transmission of HIV. The mother was given one tablet of 200 mg at the onset of labour and the neonate was given 2 mg/kg body weight within 72 hours of birth. Of the 1,322 HIV positive deliveries, 1,212 (91.7%) mother-neonate pairs received nevirapine, varying from 2 to 224 at the 16 PMTCT centres, representing 80.9% to 100% of the HIV positive deliveries. Nevirapine was not administered in 110 (8.3%) deliveries because either the mother arrived after onset of labour, or the neonate was assessed not fit to be administered nevirapine, or it was a still birth, or the mother was infected with HIV-2.

**Table 2:** Hospital deliveries, medical termination of pregnancy (MTP) and nevirapine (NVP) administered in PMTCT centres in the 2005–2006 fiscal year.

**PMTCT centre serial number**	**Total number of deliveries**	**Number (%) of HIV positive deliveries**	**Number of HIV positive MTPs**	**Ratio of MTPs to HIV positive deliveries**	**Number of mother-neonate pairs given NVP**	**% mother-neonate pairs given NVP of HIV positive deliveries**
**At hospitals attached to medical college**						

2	6235	40 (0.64)	13	0.33	38	95.0
5	6052	242 (4.00)	48	0.20	224	92.6
7	4007	134 (3.34)	24	0.18	121	90.3
8	6884	70 (1.02)	8	0.11	64	91.4
9	9258	215 (2.32)	10	0.05	203	94.4
12	8696	76 (0.87)	6	0.08	66	86.8
13	18336	157 (0.86)	34	0.22	127	80.9
15	8823	69 (0.78)	14	0.20	67	97.1

**Sub-total**	**68291**	**1003 (1.47)**	**157**	**0.16**	**910**	**90.7**

**At hospitals not attached to medical college**						

1	3239	44 (1.36)	2	0.05	42	95.5
3	3177	2 (0.06)	0	0.00	2	100
4	2458	54 (2.20)	28	0.52	52	96.3
6	1229	36 (2.93)	2	0.06	36	100
10	2449	13 (0.53)	0	0.00	13	100
11	4188	103 (2.46)	6	0.06	98	95.1
14	3432	62 (1.81)	5	0.08	54	87.1
16	2849	5 (0.18)	0	0.00	5	100

**Sub-total**	**23021**	**319 (1.39)**	**43**	**0.13**	**302**	**94.7**

**Total**	**91312**	**1322 (1.45)**	**200**	**0.15**	**1212**	**91.7**

The PMTCT centres also provide the option of medical termination of pregnancy (MTP) to HIV positive women during the first trimester of pregnancy. In the 2005–2006 fiscal year, the number of women opting for MTP was 200 at the 16 PMTCT centres (Table 2). The average ratio of the number of MTPs to the number of HIV positive deliveries was 0.15, varying from 0 to 0.52. The proportion of HIV positive mother-neonate pairs who received nevirapine and the ratio of MTPs to HIV positive deliveries were similar in PMTCT centres situated in hospitals attached to a medical college and those not attached to a medical college (Table 2).

The PMTCT centres also provide counselling and testing services to the spouse or partner of HIV positive pregnant women. Out of 2,060 HIV positive pregnant women who received post-HIV-test counselling, 1,075 (52.2%) got their spouse/partner tested of which 785 (73%) were HIV positive (Table 3). These proportions were similar in PMTCT centres situated in hospitals attached to a medical college and those not attached to a medical college.

**Table 3:** Services provided by PMTCT centres to spouse/partners of HIV positive women in the 2005–2006 fiscal year.

**PMTCT centre serial number**	**Number of HIV positive post-test counselled pregnant women**	**Number (%) of spouse/partners tested for HIV and counselled**	**Number (%) of spouse/partners HIV positive**
**At hospitals attached to medical college**			

2	77	61 (79.2)	53 (86.9)
5	321	161 (50.2)	125 (77.6)
7	100	62 (62.0	49 (79.0)
8	124	57 (46.0)	46 (80.7)
9	341	99 (29.0)	74 (74.7)
12	136	77 (56.6)	46 (59.7)
13	253	146 (57.7)	96 (65.8)
15	108	70 (64.8)	51 (72.9)

**Sub-total**	**1460**	**733 (50.2)**	**540 (73.7)**

**At hospitals not attached to medical college**			

1	80	44 (55.0)	23 (52.3)
3	17	10 (58.8)	7 (70.0)
4	136	93 (68.4)	74 (79.6)
6	77	37 (48.1)	24 (64.9)
10	36	14 (38.9)	14 (100)
11	116	72 (62.1)	52 (72.2)
14	109	62 (56.9)	41 (66.1)
16	29	10 (34.5)	10 (100)

**Sub-total**	**600**	**342 (57.0)**	**245 (71.6)**

**Total**	**2060**	**1075 (52.2)**	**785 (73.0)**

The total economic cost of the 16 PMTCT centres in the 2005–2006 fiscal year ranged from INR 358,349 (US$ 8,095) to INR 2,263,496 (US$ 51,129) with a mean of INR 773,385 (US$ 17,470) and a median of INR 642,471 (US$ 14,513) (Table 4). Personnel costs accounted for 47.3% of the total economic cost (range 41.2–62.1%), recurrent goods 31.7% (range 19.3–42.4%), rentals 13.2% (range 8.7–20.3%), recurrent services 6.3% (range 2.4–12.9%) and capital goods 1.5% (range 0.8–2.4%). The total economic cost of the 8 PMTCT centres situated in hospitals attached to a medical college averaged INR 1,082,390 (US$ 24,450) whereas that of the 8 PMTCT centres situated in hospitals not attached to a medical college averaged INR 464,379 (US$ 10,490). The financial cost of all 16 PMTCT centres combined was 70.3% of the economic cost; this was slightly higher for PMTCT centres situated in hospitals attached to a medical college (72.4%) versus those not attached to a medical college (65.3%).

**Table 4:** Economic cost of PMTCT centres in the 2005–2006 fiscal year.

**PMTCT centre serial number**	**Total economic cost (INR)**	**Total economic cost (USD)**	**Economic cost (%)**				
			
			**Personnel**	**Recurrent goods**	**Recurrent services**	**Capital goods**	**Rentals**
**At hospitals attached to medical college**							

2	902383	20384	42.8	33.5	10.1	1.5	12.1
5	1162988	26270	41.2	39.0	8.5	1.7	9.6
7	828229	18709	45.5	27.8	12.8	1.8	12.2
8	679994	15360	46.1	39.6	3.4	1.9	9.0
9	998853	22563	43.0	42.4	3.6	0.9	10.2
12	875222	19770	49.7	38.3	2.4	1.0	8.7
13	2263496	51129	47.4	25.6	5.7	1.8	19.5
15	947957	21413	42.4	32.2	8.9	1.2	15.4

**Sub-total**	**8659122**	**195598**	**45.0**	**33.5**	**6.8**	**1.5**	**13.3**

**At hospitals not attached to medical college**							

1	455197	10282	54.3	28.6	4.6	1.4	11.1
3	358349	8095	62.1	23.3	4.6	1.4	8.7
4	501213	11322	54.4	29.4	3.1	1.4	11.8
6	558155	12608	56.4	19.3	2.7	1.3	20.3
10	422372	9541	56.6	22.8	3.2	2.4	15.0
11	426687	9638	43.1	40.6	4.4	2.4	9.5
14	604947	13665	41.3	30.5	12.9	0.8	14.6
16	388110	8767	59.1	26.2	3.5	1.8	9.4

**Sub-total**	**3715030**	**83918**	**52.7**	**27.6**	**5.2**	**1.6**	**13.0**

**Total**	**12374152**	**279516**	**47.3**	**31.7**	**6.3**	**1.5**	**13.2**

INR is Indian Rupee.

US$ 1 = INR 44.27 (average exchange rate for fiscal year April 2005 – March 2006) [10]

**Figure 1:**
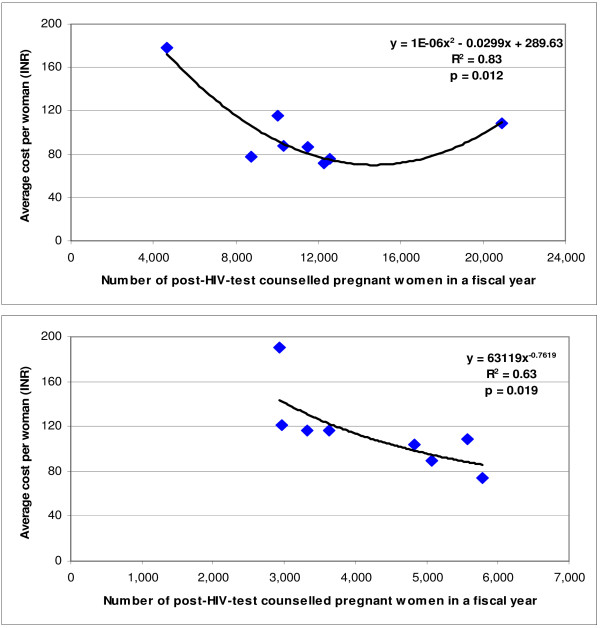
Relation between scale and cost per post-HIV-test counselled pregnant woman for PMTCT centres at hospitals attached to a medical college (upper) and those not attached to a medical college (lower) in the 2005–2006 fiscal year.

The average economic cost per post-HIV-test counselled pregnant woman for the 16 PMTCT centres was INR 98.9 (US$ 2.23), ranging from INR 71.4 (US$ 1.61) to INR 189.9 (US$ 4.29). This average economic cost was 14.4% higher at PMTCT centres situated in hospitals not attached to a medical college (INR 108.9, US$ 2.46) as compared with those attached to a medical college (INR 95.2, US$ 2.15). The relation between scale and unit economic cost was mostly inverse with a suggestion of an upward inflection at the highest end of the scale for PMTCT centres situated in hospitals attached to a medical college, with the best fit obtained using a quadratic function (Figure 1). This relation was inverse without an upward inflection for PMTCT centres not attached to a medical college, with the best fit obtained using a power function (Figure 1). If all 16 PMTCT centres were consider together, the best fit for this relation was obtained using a quadratic function (R^2 ^= 0.42, p = 0.03).

**Figure 2:**
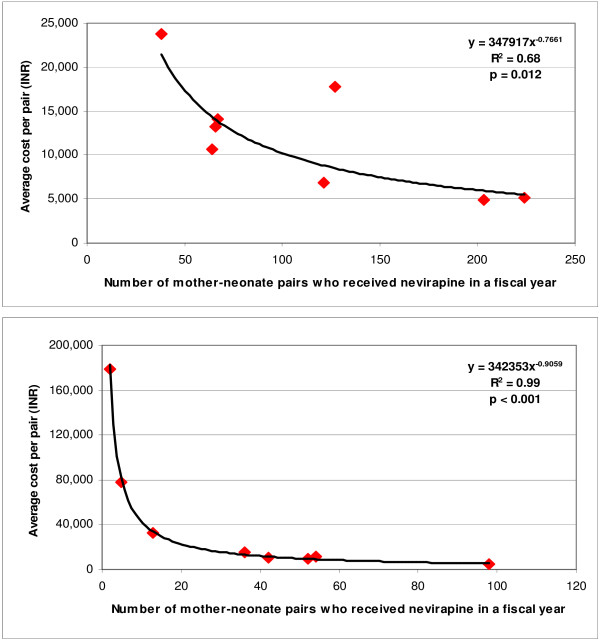
Relation between scale and cost per mother-neonate pair who received nevirapine for PMTCT centres at hospitals attached to medical college (upper) and those not attached to a medical college (lower) in the 2005–2006 fiscal year.

**Figure 3:**
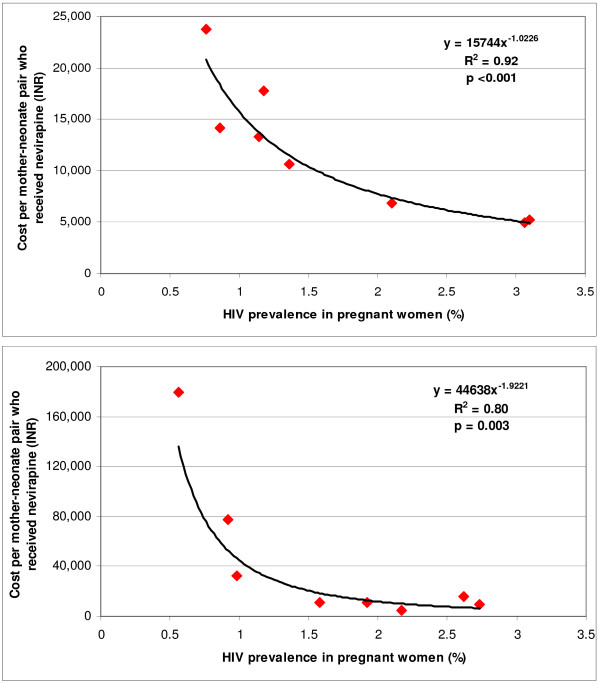
Relation between HIV prevalence among pregnant women and cost per mother-neonate pair who received nevirapine for PMTCT centres at hospitals attached to medical college (upper) and those not attached to a medical college (lower) in the 2005–2006 fiscal year.

The average economic cost per mother-neonate pair who received nevirapine for the 16 PMTCT centres was INR 10,210 (US$ 231), ranging 41-fold from INR 4,354 (US$ 98) to INR 179,175 (US$ 4,047). This average economic cost was 29.3% higher for the relatively smaller PMTCT centres situated in hospitals not attached to a medical college (INR 12,301, US$ 278) as compared with the larger PMTCT centres situated in hospitals attached to a medical college (INR 9,516, US$ 215). There was a significant inverse relation between scale and unit economic cost, with the best fit obtained using a power function for PMTCT centres situated in hospitals attached to a medical college as well as those not attached to a medical college (Figure 2). If all 16 PMTCT centres were consider together, the best fit for this relation was also obtained using a power function (R^2 ^= 0.87, p < 0.001). There was also a significant inverse relation between HIV prevalence among pregnant women and economic cost per mother-neonate pair who received nevirapine, with the best fit obtained using a power function for both the PMTCT centres situated in hospitals attached to a medical college and those not attached to a medical college (Figure 3). The two PMTCT centres that had extremely high unit cost per mother-neonate pair who received nevirapine (INR 179,175 [US$4,047] and INR 77,622 [US$ 1,753]) had HIV prevalence among pregnant women less than 1% and a relatively low total number of pregnant women who received PMTCT services, resulting in only 2 and 5 mother-neonate pairs receiving nevirapine in a fiscal year (centres 3 and 16 in Tables 1 and 5). Both these centres were situated in hospitals not attached to a medical college.

**Table 5:** Efficiency of PMTCT centres in the 2005–2006 fiscal year.

**PMTCT centre serial number**	**Economic cost per post-HIV-test counselled pregnant woman**		**Economic cost per mother-neonate pair receiving nevirapine**	
	
	**INR**	**US$**	**INR**	**US$**
**At hospitals attached to medical college**				

2	87.3	1.97	23747	536
5	115.8	2.62	5192	117
7	177.8	4.02	6845	155
8	77.9	1.76	10625	240
9	86.9	1.96	4920	111
12	71.4	1.61	13261	300
13	108.3	2.45	17823	403
15	75.6	1.71	14149	320

**Average**	**95.2**	**2.15**	**9516**	**215**

**At hospitals not attached to medical college**				

1	89.6	2.02	10838	245
3	120.9	2.73	179175	4047
4	103.7	2.34	9639	218
6	189.9	4.29	15504	350
10	116.3	2.63	32490	734
11	73.9	1.67	4354	98
14	108.4	2.45	11203	253
16	116.7	2.64	77622	1753

**Average**	**108.9**	**2.46**	**12301**	**278**

**Average for all**	**98.9**	**2.23**	**10210**	**231**

INR is Indian Rupee.

US$ 1 = INR 44.27 (average exchange rate for fiscal year April 2005 – March 2006) [10]

## Discussion

This study of public sector PMTCT centres in the Indian state of Andhra Pradesh reveals that these centres provide counselling and testing services to pregnant women, administer nevirapine to HIV positive mothers and neonates, provide HIV positive pregnant women with the option of medical termination of pregnancy if they are still in the first trimester of pregnancy, and provide counselling and testing to the spouse or partner of HIV positive women.

The relatively modest 5–7% pregnant women not receiving post-HIV-test counselling is related to the practice of using rapid tests and handing over results on the same day. During post-HIV-test counselling, the HIV positive pregnant women are advised to bring their spouse/partners for counselling. About half of the HIV positive pregnant women brought their spouse/partners for testing. Of the spouses/partners tested, 73% were found positive. Further efforts to increase the proportion of spouses/partners counselled and tested would be useful.

The relation between unit economic cost and scale was mostly inverse. An upward inflection was observed at the highest end of the scale for post-test-counselled pregnant women at large PMTCT centres situated in hospitals attached to a medical college, which suggested that the unit cost may start increasing beyond about 14,000 post-test-counselled pregnant women in a year. This however should be interpreted with caution as the upward inflection was only due to one data point. Such an upward inflection at the higher end of the scale has also been suggested in data from voluntary counselling and testing (VCT) centres and sex worker programmes for HIV prevention in this Indian state [11,12]. We have recently reported a predominantly inverse relation between unit economic cost and scale for over 200 HIV prevention programs of various types studied as part of the PANCEA study in five low- and middle-income countries [13].

The unit cost for mother-neonates who received nevirapine varied very widely between PMTCT centres, over 40-fold. The extremely high unit costs were found in two PMTCT centres situated in relatively small hospitals not attached to a medical college that provided nevirapine to only 2 and 5 mother-neonate pairs in a fiscal year because of relatively low HIV prevalence and total number of pregnant women served. This has particular significance for further planning of PMTCT services in Andhra Pradesh, as new services are being initiated at relatively smaller health facilities where the number of HIV positive mother-neonate pairs may not be high. Importantly, since April 2006, the government has started over 350 integrated counselling and testing centres (ICTCs) at relatively smaller community health centres and primary health centres in Andhra Pradesh to provide both VCT and PMTCT services at one place. In the background of poor efficiency of isolated PMTCT services found in our study at smaller facilities with relatively low HIV prevalence, the new approach of combining VCT and PMTCT services seems justified. This approach is likely to enhance efficiency at smaller facilities but would have to be studied for proper understanding of how this can yield optimal advantages. This integration of services approach is now recommended by India's National AIDS Control Organization and is also being applied to larger health facilities where VCT and PMTCT services existed earlier, combining them under the umbrella of ICTCs [14]. Where applicable, linkage of ICTCs with other related services such as those for tuberculosis and sexually transmitted infection as well as HIV/AIDS treatment and care, are also being attempted. These efforts are in an early stage and would have to be monitored as they evolve.

The relation between unit cost and scale reported in this paper for PMTCT and that reported earlier for VCT by us [7], and our recent analysis of the changing cost of VCT in Andhra Pradesh over a period of three years [12], can usefully inform further planning of PMTCT and VCT services in this India state. It would also be useful to subsequently compare the outputs and efficiency of the new ICTC approach with our current findings to assess the advantages that may accrue with the new integrated approach. PMTCT is receiving increasing attention globally as an important component of HIV control [15]. Cost-effectiveness data have been reported for PMTCT from other parts of the world, mainly sub-Saharan Africa [16-19]. The efficiency data reported by us could form the basis of cost-effectiveness calculations in India if supplemented with additional data and analyses, which could further inform decisions about how best to use the available resources for HIV control in India.

## Conclusion

The inverse relation between unit cost and scale for prevention of mother to child transmission of HIV services, and the extremely high unit cost at some centres providing nevirapine to a very small number of mother-neonate pairs annually, needs to be taken into account for further planning of services in this Indian state for prevention of mother to child transmission of HIV. Combining PMTCT and VCT services would be the preferred approach to increase efficiency, particularly at relatively smaller centres.

## Competing interests

The author(s) declare that they have no competing interests.

## Authors' contributions

LD led the study and guided the design, data collection, analysis, and interpretation. SGPK, YKR, and MCR contributed to the design, data collection, analysis and interpretation. EM and JGK contributed to the design of the instruments and interpretation. RD guided the analysis and interpretation. LD, SGPK and RD drafted the paper in the form finally presented, and all authors read and approved the final version of the paper except YKR who unfortunately passed away before the final version.

## Pre-publication history

The pre-publication history for this paper can be accessed here:


